# *Clonorchis sinensis* Granulin Promotes Malignant Transformation of Hepatocyte Through EGFR-Mediated RAS/MAPK/ERK and PI3K/Akt Signaling Pathways

**DOI:** 10.3389/fcimb.2021.734750

**Published:** 2021-11-10

**Authors:** Caiqin Wang, Qing He, Yingxuan Yin, Yinjuan Wu, Xuerong Li

**Affiliations:** ^1^ Department of Medical Oncology, Guangdong Institute of Gastroenterology, Guangdong Provincial Key Laboratory of Colorectal and Pelvic Floor Diseases, The Sixth Affiliated Hospital of Sun Yat-sen University, Guangzhou, China; ^2^ Department of Parasitology, Zhongshan School of Medicine, Sun Yat-sen University, Guangzhou, China; ^3^ Key Laboratory for Tropical Diseases Control of Ministry of Education, Sun Yat-sen University, Guangzhou, China; ^4^ Provincial Engineering Technology Research Center for Biological Vector Control, Guangzhou, China; ^5^ China Atomic Energy Authority (CAEA) Center of Excellence on Nuclear Technology Applications for Insect Control, Beijing, China

**Keywords:** *Clonorchis sinensis* granulin (*Cs*GRN), hepatocyte, malignant transformation, RAS/MAPK/ERK signaling pathways, PI3K/Akt signaling pathways

## Abstract

The biological functions of growth factor, such as granulins, have been explored in parasites, and we elucidated that *Clonorchis sinensis* granulin (*Cs*GRN) promoted the metastasis of hepatocellular carcinoma in our previous study. However, it is still unclear for the malignant transformation role of *Cs*GRN in normal human hepatocytes. In this study, by transfecting pEGFP-C1-*Cs*GRN eukaryotic expression plasmid, a cell line with stable overexpression of *Cs*GRN in normal hepatocyte (LO2-GRN cells) was constructed. The effects on cell proliferation were detected by carrying out cell counting kit-8 (CCK8) assay and colony formation assay. Additionally, we conducted flow cytometry analysis to determine whether the proliferation of *Cs*GRN was due to cell cycle arrest. Subsequently, the migration ability and the invasion ability of LO2-GRN cells were evaluated through wound-healing assay and transwell assay. Meanwhile, the levels of the markers of RAS/MAPK/ERK and PI3K/Akt signaling pathways activation in LO2-GRN cells were assessed by quantitative RT-PCR and Western blot. Our results indicated that *Cs*GRN promoted the proliferation of LO2 cells by regulating the expression of cell-cycle-related genes. Moreover, the overexpression of *Cs*GRN regulates malignant metastasis of liver cells by inducing the upregulation of epithelial–mesenchymal transition (EMT) marker proteins. Furthermore, both mRNA and protein expression levels of p-EGFR, RAS, p-ERK, p-AKT, p-PI3K, and p-braf have been enhanced by *Cs*GRN. These results showed that *Cs*GRN promoted the malignant transformation of hepatocytes by regulating epidermal growth factor receptor (EGFR)-mediated RAS/MAPK/ERK and PI3K/Akt signaling pathways, which suggested that *Cs*GRN could serve as a novel oncoprotein during *Clonorchis sinensis*–associated malignant transformation of hepatocytes.

## Introduction

*Clonorchis sinensis* chronic infection is concerned with the outcomes of various pathologic processes, including hepatic fibrosis, liver cirrhosis, carcinogenesis, etc. (Rim, 2005; Marcos et al., 2008; Hong and Fang, 2012). The majority of studies demonstrated that *Clonorchiasis* is mostly prevalent in China, Vietnam, and Korea. Approximately 13 million people are infected by *Clonorchis sinensis* in China, followed by the estimation of 1 million in Vietnam and 1.2 million in Korea (Na et al., 2020; Qian et al., 2020). *Clonorchis sinensis* has been classified as a class I carcinogen by the International Agency for Research on Cancer and is well-known to be recognized as a risk factor for cholangiocarcinoma (CCA) (Bouvard et al., 2009; Qian et al., 2012). What’s more, some epidemiological and clinical studies showed that the incidence of hepatocellular carcinoma (HCC) in patients with *Clonorchis sinensis* infection is much higher than that in non-infected patients, which indicated that *Clonorchis sinensis* may be a significant risk factor for HCC (Kim, 1984; Tan et al., 2008).

Mechanical obstruction of bile duct by worms, physical damage to bile duct epithelium by feeding and migration of worms, and chemical stimulation of excretion and secretion products (ESPs) are the main pathogenic mechanisms of *Clonorchis sinensis* infection (Qian et al., 2016; Kim et al., 2016). ESPs of *Clonorchis sinensis* (*Cs*ESPs) have been proven to promote the proliferation and migration of HCC, and inhibit their apoptosis (Chen et al., 2013; Chen et al., 2015). In our previous studies, *Clonorchis sinensis* granulin (*Cs*GRN), as a component of *Cs*ESPs, was observed in hepatocytes and biliary ducts of the *Balb/c* mice that were infected with *Clonorchis sinensis*. The phylogenetic analysis illustrated that granulin of *C. sinensis* was particularly close to that of *Opisthorchis viverrini*, which demonstrated that *Cs*GRN may regulate cell division, survival, movement, and migration like *Opisthorchis viverrini* granulin 1 (*Ov* -GRN-1), a paralogue of progranulin (PGRN) secreted by *O. viverrine* (Smout et al., 2009; Bansal et al., 2017; Wang et al., 2017; Haugen et al., 2018). It has been reported that granulin played a vital role in the carcinogenesis of a series of malignancies (Bateman and Bennett, 2009). Most of members of the granulin family proteins are proved to have similar functions to growth factors. They bind to a variety of cell surface proteins and directly or indirectly participate in different signaling pathways to regulate cell proliferation, apoptosis, invasion, and metastasis (Yung et al., 2015; Marsh et al., 2016; Pan et al., 2018). For instance, liver fluke granulin stimulated cell proliferation, wound healing, and angiogenesis *in vitro*, and it is involved in the pathogenesis of *Opisthorchis viverrini in vivo* (Arunsan et al., 2020). The overexpression of PGRN in tumor cells can promote cell differentiation, invasion, and metastasis; resist apoptosis; and reduce the sensitivity to anticancer drugs (He and Bateman, 1999; Jones et al., 2003). In addition, taking advantage of granulin targeting could serves as a potential therapeutic strategy to convert pancreatic ductal adenocarcinoma (PDAC) into metastatic tumors by restoring CD8^+^ T cell infiltration (Quaranta et al., 2018). According to the sequence analysis, *Cs*GRN has 28% overall identity with human PGRN, suggesting that *Cs*GRN may be involved in mediating tumor progression (Yung et al., 2015; Marsh et al., 2016). Moreover, the overexpression of recombinant *Cs*GRN could promote cell migration and invasion by inducing liver epithelial–mesenchymal transition (EMT) *via* the ERK and PI3K/AKT signal pathways in HCC cell line PLC cells, which indicated that *Cs*GRN was involved in the pathogenesis of HCC (Young et al., 2010; Wang et al., 2011; Wang et al., 2017). Nevertheless, further investigation on how *Cs*GRN influences the malignant transformation of normal hepatocyte is still required.

In the present study, we aimed to evaluate the contribution of granulin from *Clonorchis sinensis* (*Cs*GRN) to the malignant transformation of hepatocyte LO2 cells. Recombinant plasmid of *Cs*GRN was constructed and was stably overexpressed in LO2 cells. In addition, its influence on the malignant proliferation and metastasis of LO2 cells were investigated by using cell counting kit 8 (CCK8) assay and transwell assay, respectively. What’s more, both quantitative RT-PCR (q-PCR) and Western blot were used to explore the mechanisms of HCC carcinogenesis induced by *Cs*GRN.

## Materials and Methods

### Inhibitors

EGFR inhibitor: Gefitinib (No. #S1025); PI3K inhibitor: Buparlisib (No. #S2247); Akt inhibitor: Perifosine (No. #S1037); braf inhibitor: Sorafenib (BAY 43-9006) tosylate (No. #S1040); ERK inhibitor: Temuterkib (No. #S8534). All above inhibitors were bought from Selleck (USA). RAS inhibitor, RAS GTPase inhibitor 1 (No. # T12692), was purchased from Topscience (China).

### Cell Culture

Human normal hepatocyte LO2 cells were cultured in DMEM medium (Gibco, Carlsbad, USA) supplemented with 10% fetal bovine serum (FBS; Gibco) with 95% O_2_ and 5% CO_2_ at 37°C. The cell lines were gifted from the Center of Hepato-Pancreato-Biliary Surgery, the First Affiliated Hospital of Sun Yat-sen University.

### Construction and Stable Transfecting of Recombinant Granulin

pEGFP-C1-*Cs*GRN recombinant plasmid and pEGFP-C1 vector (Promega) were transfected into human liver cell lines LO2 (*Cs*GRN-LO2), respectively, as our previously described (Wang et al., 2017). The cells stably expressing EGFP-tagged *Cs*GRN were selectively established by 400 μg/ml G418 and identified by fluorescence microscope and qRT-PCR analysis.

### Recombinant *Cs*GRN Genes Cloned and Purified

The methods of gene cloning and purification of recombinant *Cs*GRN were used as our previous study (Wang et al., 2017). Briefly, the ORF of *Cs*GRN (GenBank KY855531) has a genome length of 714 bp and was cloned into the pMAL-c2x vector (preserved in our lab). Following digestion with restriction enzymes, the plasmid DNA was attached to the pET-28a (+) expression vector (Novagen, Darmstadt, Germany), before being introduced into the *E. coli* BL21 (DE3) (Promega). Then, we induced selected clones at 20°C for 12–18 h with 1 mM IPTG (Sigma, Guangzhou, China) following growth. By using the Amylose Resin-Bind Purification Kit (BioLabs), we purified recombinant protein (MBP-GRN) from the supernatant.

### Quantitative RT-PCR

Total RNA was obtained by using Trizol reagent (Invitrogen, Carlsbad, CA, USA), and the total cDNA was generated using ABM’s 5**×** All-In-One RT Master Mix (Transgen, Beijing, China). SYBR Premix Ex Taq (Takara, Dalian, China) was used for quantitative analysis of the expression of mRNA level of indicated gene. PCR quantification was performed using the CFX96 Real-Time PCR system (Bio-Rad). GAPDH was performed as the reference gene. Following are the formulae used to calculate relative mRNA expression: ΔCt = Ct (sample) − Ct (GAPDH), ΔΔCt (sample) = ΔCt (sample) −ΔCt (calibrator). The relative quantification of mRNA was used to calculate the fold change. cDNA was amplified using specific primers (**Supplementary Table**).

### Measurement of Cell Proliferation

Cell proliferation was evaluated by Cell Counting Kit 8(CCK8) assay as well as Colony Formation assay, respectively. Briefly, cells were seeded onto 96-well plates with 8×10^3^/100 µl, after 24 h, replaced with serum-free medium 100 µl added with 10 µl CCK8 in each well, and then placed in the incubator for 30 min to 3 h, and the absorbance value of 450 nm wavelength was measured by microplate reader. The long-term effects of *Cs*GRN on LO2 cell proliferation were analyzed with a colony formation assay. Cells were seeded into six-well plates and cultured for 2–3 weeks. Medium was renovated every 3 days. After washing with phosphate buffer saline (PBS) for three times, the cells were then fixed with 4% paraformaldehyde, stained with crystal violet solution, and washed with ddH_2_O to remove the excess dye. The colony number was analyzed with Image J software.

### Cell Cycle

Cell cycle was evaluated by flow cytofluorometry. Briefly, cells were seeded into six-well plates at a density of 10^6^ cells per well in completed medium. After 24 h, the cells were stained with Propidium iodide [MultiSciences (Lianke) Biotech Co, Ltd.] and analyzed by flow cytometry (Coulter Beckman Gallios) and analyzed with Flowjo software.

### Transwell Assay

The invasion ability of *Cs*GRN-LO2 cells was evaluated though Transwell invasion chambers (Costar, NY, USA). Transfected *Cs*GRN-LO2 cells cultured in 24-well plates (1×10^6^cells/ml) with non-serum medium and Matrigel (BD Biosciences, Heidelberg, Germany) mixed on the upper chambers (1:8), while medium containing 10% FBS was added into the lower chambers for 24 h at 37°C. The invading cells were then stained with 1% crystal violet (Beyotime Biotechnology, Guangzhou, China) for 15 min, followed by imaging with light microscope (Leica DMI3000B, Wetzlar, Germany) and counting the numbers of migrated cells in five random fields. The cell motility points were measured by using Image J software.

### Wound Healing Assay

The effects of *Cs*GRN on LO2 cells migration were analyzed with a wound-healing assay. Transfected LO2 cells were moved into six-well plates and hatched for 24 h. When cells were grown to 80%, the bottom of plate was scratched with a 200 μl pipette tip, followed by observing and imaging with light microscope (Leica DMI3000B, Wetzlar, Germany) every 24 h for 72 h.

### Western Blot

Cells were dissolved by RIPA buffer (Beyotime, Shanghai, China) and quantified by BCA Protein Assay (Trans, Beijing, China). Thirty μg total proteins were separated by SDS-PAGE, followed by transferring onto PVDF membranes (Whatman, Maidstone, UK) and then sealed with 5% skimmed milk in TBST for 2 h at room temperature. After incubation in primary and secondary antibodies, the blots were detected by ECL (Millipore, Billerica, USA). The following antibodies were used: anti-MBP tag monoclonal antibody (1:2,000, Novagen), rat anti-*Cs*GRN serum (1:200), anti-E-cadherin (1:2,000), anti-vimentin (1:2,000), anti-N-cadherin (1:2,000), anti-ZO-1 (1:2,000), anti-β-catenin (1:2,000), anti-p18 (1:2,000), anti-p21 (1:2,000), anti-p27 (1:2,000), anti-CDK2 (1:2,000), anti-CDK4 (1:2,000), anti-CDK6 (1:2,000), anti-CyclinD1 (1:2,000), anti-CyclinD3 (1:2,000), p-ERK (1:2,000), ERK (1:2,000), p-AKT (1:2,000), p-EGFR (1:2,000), RAS (1:2,000), p-braf (1:2,000), and anti-GAPDH (1:2,000). All antibodies were products from Cell Signaling Technology (CST, Boston, USA). Secondary antibodies were purchased form for Proteintech Group: goat anti-mouse IgG-HRP (1:5,000) and goat anti-rabbit IgG-HRP (1:5,000). For quantification of western blot images, ImageJ (https://imagej.nih.gov/ij/index.html) was used.

### Statistical Analysis

Statistical analysis was performed using SPSS26.0 statistical software. Student’s *t-*test and ANOVA were used to analyze statistical differences. Data were shown as means ± S.D., and significance was described as *P* < 0.05.

## Results

### *Cs*GRN Overexpressed in LO2-GRN Cell

**Figure 1:**
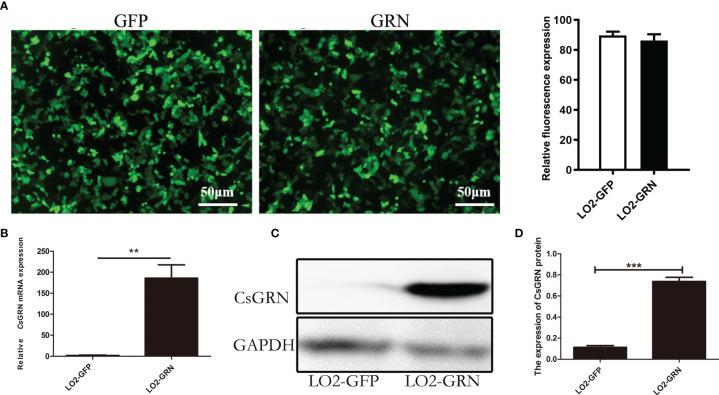
Stable expression of pEGFP-C1-*Cs*GRN in human LO2 hepatocytes. LO2 hepatocytes were stably transfected with pEGFP-C1 or pEGFP-C1-*Cs*GRN and selected with G418-containing medium. Cell lines were named as LO2-GFP and LO2-GRN, respectively. **(A)** Green fluorescence was observed by inverted microscope to determine the efficiency of transfection (scale bar is 50 µm), and the quantification of green fluorescence was measured by Image J software. **(B)** Relative mRNA levels of *Cs*GRN were quantified by q-PCR. **(C)** The expression of *Cs*GRN protein were determined by Western blotting. **(D)** Relative protein levels of *Cs*GRN were quantified by Image J software from three independent experiments. ^**^ *P* < 0.01 *versus* control group, ^***^ *P* < 0.001 *versus* control group.

To determine the potential role of *Cs*GRN overexpression on inducing the malignant transformation of human hepatocytes, an LO2 cell line with stable expression of EGFP-Tagged *Cs*GRN fusion protein was constructed, while empty vector transfected LO2 cells were used as negative controls. Under the inverted microscope, the green fluorescence was emitted by LO2 cells that were transformed with pEGFP-C1 and pEGFP-C1-*Cs*GRN (**Figure 1A**). Both Western blot and qPCR revealed the higher expression of *Cs*GRN in the stably transfected LO2 cells (**Figures 1B–D**). The eukaryotic expressing vector of pEGFP-C1 is successfully constructed (named as LO2-GFP), and the LO2 cell line that stably expresses pEGFP-C1-*Cs*GRN (named as LO2-GRN) has provided solid experimental foundation for further studies.

### Overexpression of *Cs*GRN Promoted LO2 Cell Proliferation and Arrested in G2/M Phase

**Figure 2:**
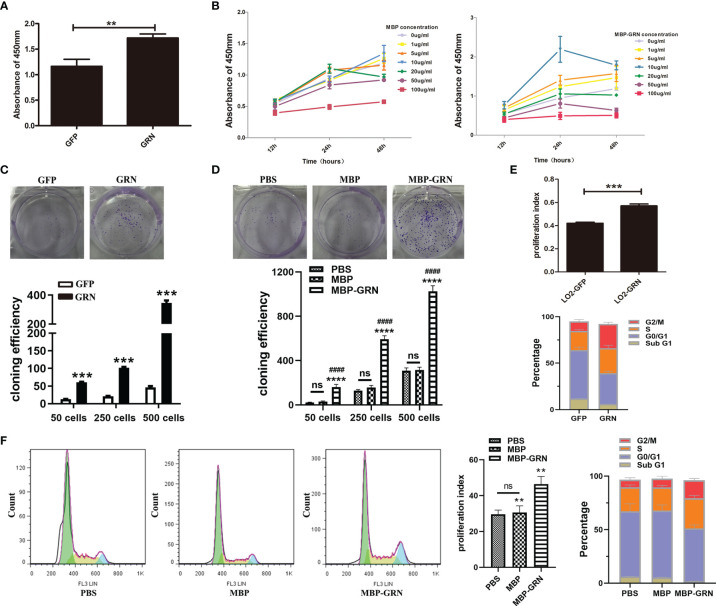
Effects of *Cs*GRN overexpression on promoting proliferation, colony formation, and cell cycle distribution of LO2 cells. **(A, B)** Cell proliferation capability of *Cs*GRN-overexpressed LO2 cells **(A)** and recombinant *Cs*GRN protein–treated LO2 cells were detected by CCK8 assay **(B)**. ^**^ *P* < 0.01 *versus* control group. **(C)** Colony formation assay was performed in *Cs*GRN-overexpressed LO2 cells. ^***^ *P* < 0.001 *versus* control group. **(D)** Colony formation assay was performed in recombinant *Cs*GRN protein–treated LO2 cells. ^****^ *P* < 0.0001 *versus* control group, ^####^ *P <* 0.0001 *versus* MBP group. **(E, F)** Cell cycle distribution was analyzed by flow cytometry. The proliferation index (PI) and the percentage of cell cycle phase in *Cs*GRN-overexpressed LO2 cells **(E)**. Histogram of the typical cell cycle, PI, and the percentage of cell cycle phase in recombinant *Cs*GRN protein–treated LO2 cells **(F)**. ^**^ *P* < 0.01 *versus* control group, ^***^ *P* < 0.001 *versus* control group. The data of proliferation index (PI) were calculated by the formula: PI = (S + G2M)/(G0/1 + S + G2M). NS means NO statistic difference.

Next, we explored the proliferation and cell cycle of LO2 cells. We used two cell models: *Cs*GRN-overexpressed LO2 cells and recombinant *Cs*GRN protein (called as MBP-GRN) treated-LO2 cells to simulate secreted granulin of *Clonorchis sinensis* in the liver. Compared with control cells, *Cs*GRN-overexpressed and recombinant *Cs*GRN protein treated-LO2 cells both grew quickly and formed more cell clones (**Figures 2A–D**). Since *Cs*GRN induced hepatocyte cell growth, we further used flow cytometry analysis to determine whether the proliferation of *Cs*GRN was due to cell cycle arrest. LO2-GRN cells and MBP-GRN cultured-LO2 cells showed an increased proliferation index with a significant amount of cell accumulation at both S and G2/M phase (**Figures 2E, F**), referring to that *Cs*GRN overexpression induces cell proliferation by stimulating S phase entry and G2/M phase retardation.

### *Cs*GRN Promoted LO2 Cell Proliferation by Modulating Cell Cycle Regulators

**Figure 3:**
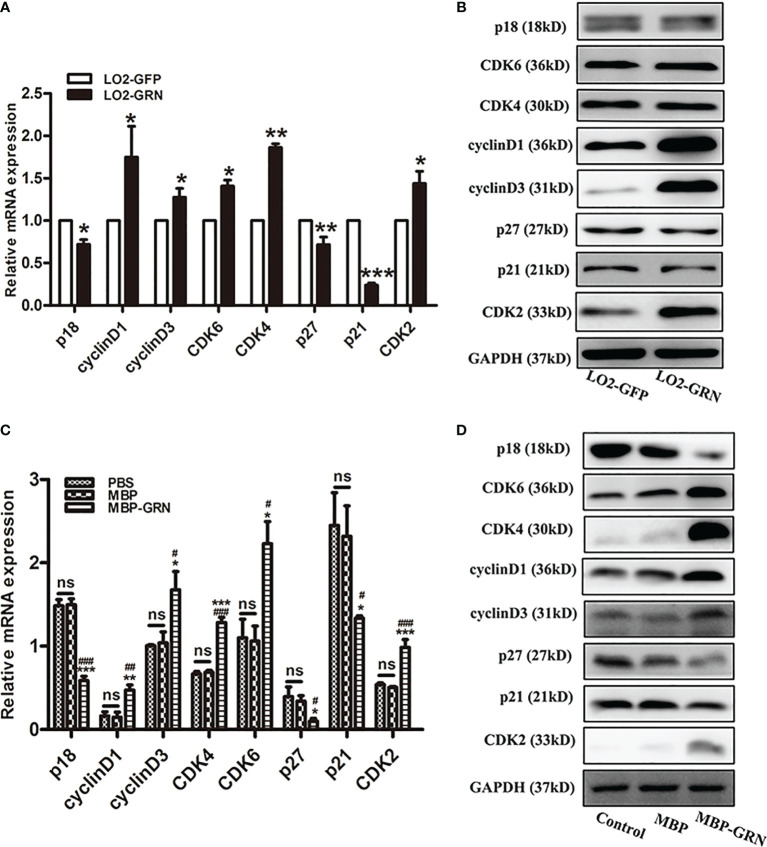
Effects of *Cs*GRN overexpression on the regulation of cell-cycle-related genes expression. **(A, B)** The mRNA and protein expression of p18, CyclinD1, CyclinD3, CDK6, CDK4, P27, P21, and CDK2 were detected in *Cs*GRN-overexpressed LO2 cells. ^*^ *P* < 0.05 *versus* control group, ^**^ *P* < 0.01 *versus* control group, ^***^ *P* < 0.001 *versus* control group. **(C, D)** The mRNA and protein expression of cell-cycle-related genes were detected in recombinant *Cs*GRN protein–treated LO2 cells. ^*^ *P* < 0.05 *versus* PBS group, ^**^ *P* < 0.01 *versus* PBS group, ^***^ *P*<0.001 *versus* PBS group. ^#^ *P <* 0.05 *versus* MBP group, ^##^ *P <* 0.01 *versus* MBP group, ^###^ *P <* 0.001 *versus* MBP group. The relative mRNA levels of the target genes were determined by q-PCR. The protein expression was determined by Western blot, and the quantification of protein expression was measured by Image J software. Three independent experiments were conducted. NS means NO statistic difference.

To investigate the possible molecular mechanism of *Cs*GRN-induced growth arrest in human hepatocyte, the mRNA and protein levels of various cell-cycle-related regulators were examined. Cell cycle regulation is a delicate biological process, and it forms a complex signaling molecular network system with the participation of multiple genes and proteins (Dynlacht, 1997). The significant increase of CyclinD1, CyclinD3, CDK2, CDK4, and CDK6, and obvious decrease of p18, p21, and p27, both in transcriptional and translational aspects, were detected in LO2-GRN cells and MBP-GRN treated-LO2 cells (**Figure 3**, **Figure S1**). Thereby, stimulating abnormal proliferation of LO2 cells through the regulation of cell-cycle-related genes resulted in malignant transformation of normal hepatocytes.

### *Cs*GRN Enhanced the Migration and Invasion of Hepatocytes

**Figure 4:**
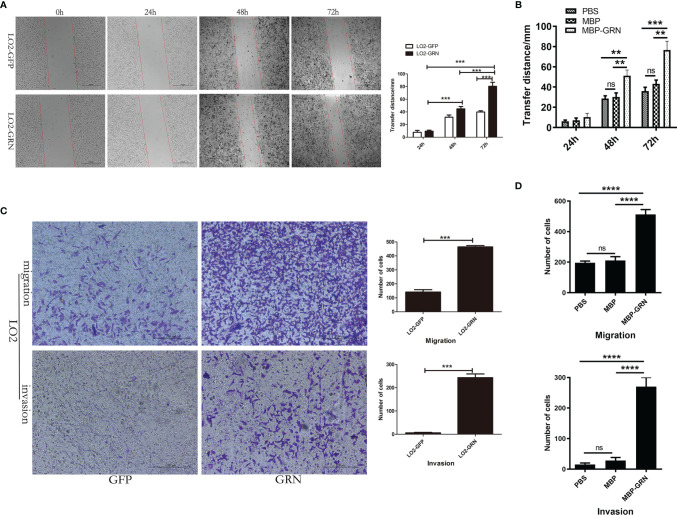
The effects of migration and invasion in *Cs*GRN overexpression LO2 cells. **(A, B)** Wound healing scratch assay was used to determine the migration of *Cs*GRN-overexpressed LO2 cells **(A)** and recombinant *Cs*GRN protein–treated LO2 cells (**B**). Dotted lines show the edges of the wound over time. **(C, D)** Transwell assay was used to determine migration and invasion of *Cs*GRN-overexpressed LO2 cells **(C)** and recombinant *Cs*GRN protein–treated LO2 cells **(D)**. Ten random fields were selected, and the migration and invasion were quantified using Image J software. ^**^ *P* < 0.01 *versus* control group, ^***^ *P* < 0.001 *versus* control group, ^****^ *P* < 0.0001 *versus* control group.

To identify the biological role of *Cs*GRN in LO2 cells, we investigated the effect of *Cs*GRN on the cell’s migration levels. The scratched wound healed quickly in LO2-GRN cells and MBP-GRN treated-LO2 cells (**Figures 4A, B**). Moreover, the effects on LO2 cells’ migration and invasion of *Cs*GRN were examined by Transwell assay. The number of transmembrane cells, both with or without Matrigel, in the LO2-GRN group and MBP-GRN cultured group were significantly elevated (**Figures 4C, D**). Together, these data suggested that *Cs*GRN promotes the migration and invasion in LO2 cells.

### *Cs*GRN Induced LO2 Cell Metastasis by Inducing EMT

**Figure 5:**
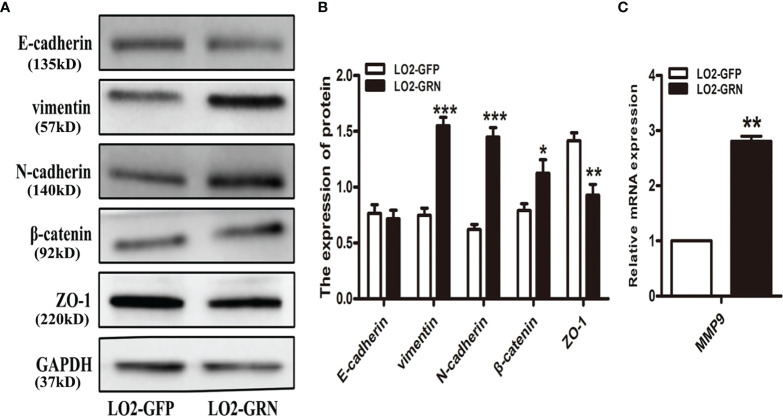
Effects of *Cs*GRN overexpression on the regulation of EMT-related gene expression. **(A)** The expression of vimentin, N-cadherin, β-catenin, and ZO-1 was detected in *Cs*GRN-overexpressed LO2 cells by Western blot. **(B)** The protein quantification was measured by Image J software. **(C)** The relative mRNA expression of matrix metalloproteinases (MMPs) was detected in transfected LO2 cells. ^*^ *P* < 0.05 *versus* control group, ^**^ *P* < 0.01 *versus* control group, ^***^ *P* < 0.001 *versus* control group.

It seems that chronically injured liver cells do not undergo epithelial-to-mesenchymal transition (EMT) (Taura et al., 2010; Chu et al., 2011; Munker et al., 2017). In contrast, it has been reported that hepatocytes in damaged liver regeneration may undergo an EMT-like process (Oh et al., 2018). EMT is considered as a significant cellular event in the process of liver fibrosis (Rowe et al., 2011). Studies revealed that more than 80% of HCC occurs when liver fibrosis or cirrhosis develops, suggesting that liver fibrosis plays an important role in the premalignant environment of the liver (Affo et al., 2017). Therefore, we investigated the possibility that *Cs*GRN affected hepatocytes’ migration and invasion by EMT. Western-blot results proved that there were significant differences between the LO2-GRN and LO2-GFP groups in the expression of vimentin, N-cadherin, β-catenin, and ZO-1 (**Figures 5A, B**). Additionally, the relative mRNA expression of matrix metalloproteinases (MMPs) in LO2-GRN cells exhibited that the MMP9 level was higher in LO2-GRN cells than that in LO2-GFP cells (**Figure 5C**). Thus, we considered that *Cs*GRN enhanced LO2 cell migration through promotion of EMT.

### *Cs*GRN Promoted Hepatocytes’ Malignant Transformation Through EGFR-Mediated RAS/MAPK/ERK and PI3K/Akt Signaling Pathways

**Figure 6:**
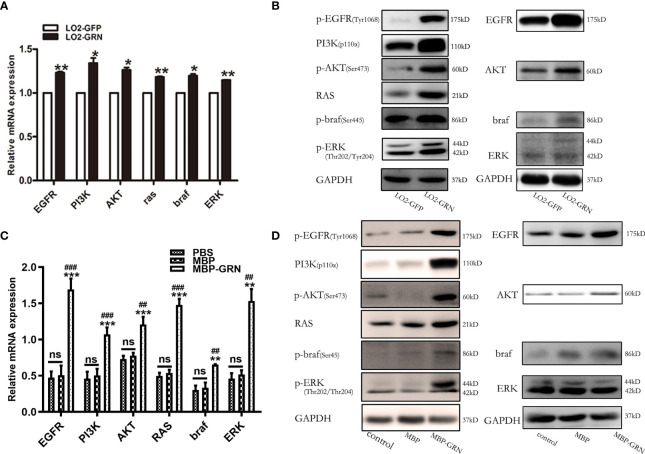
Detection of signaling pathway markers in *Cs*GRN overexpression cells. **(A, B)** The mRNA and protein levels of RAS/MAPK/ERK and PI3K/Akt signaling pathways were detected in *Cs*GRN-overexpressed LO2 cells. ^*^ *P* < 0.05 *versus* control group, ^**^ *P* < 0.01 *versus* control group. **(B, D)** The mRNA and protein levels of RAS/MAPK/ERK and PI3K/Akt signaling pathways were explored in recombinant *Cs*GRN protein–treated LO2 cells. ^**^ *P* < 0.01 *versus* PBS group, ^***^ *P* < 0.001 *versus* PBS group. ^##^ *P < *0.01 *versus* MBP group, ^###^ *P < *0.001 *versus* MBP group. The relative mRNA levels of the target genes were determined by q-PCR. The protein expression was determined by Western blot, and the quantification of protein expression was measured by Image J software. Three independent experiments were conducted.

The underlying mechanisms regulated by *Cs*GRN-induced malignant transformation of normal hepatocytes were explored. As *Cs*GRN is one member of the growth factor family, we investigated the potential downstream regulatory effects of EGFR-mediated signaling pathways. The mRNA levels of EGFR, PI3K, Akt, braf, RAS, and ERK were moderately increased both in LO2-GRN cells and MBP-GRN treated-LO2 cells (**Figures 6A, C**). While, the significant elevation of phosphorylated form of EGFR, PI3K, AKT, braf, and ERK proteins were more obvious than their non-phosphorylated form (**Figures 6B, D**). These results indicated that *Cs*GRN activated the RAS/MAPK/ERK and PI3K/Akt signaling pathways through enhancing protein phosphorylation.

**Figure 7:**
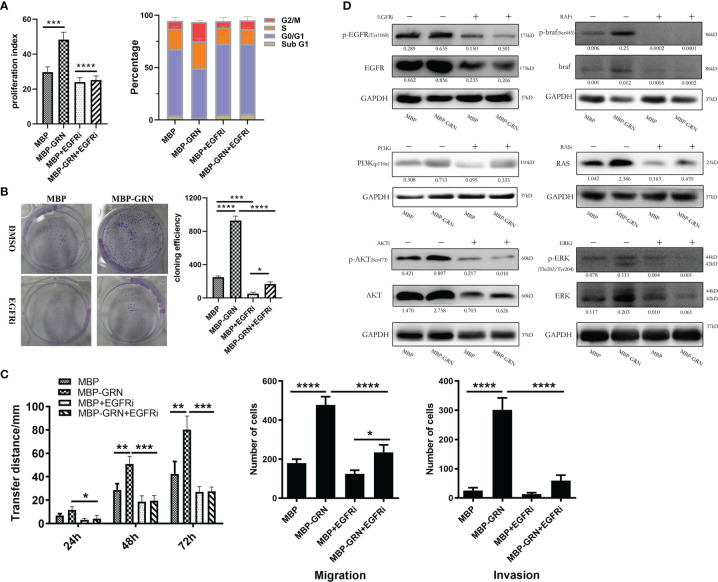
The effects and changes of RAS/MAPK/ERK and PI3K/Akt signaling pathways in LO2 cells after co-cultured with recombinant *Cs*GRN protein and EGFR inhibitor. **(A)** The histogram of PI and the percentage of cell cycle phase. **(B)** The representative images from colony formation assay and histogram of cloning efficiency. **(C)** The migration and invasion of LO2 cells were explored by wound healing scratch assay and Transwell assay. **(D)** The protein expression of RAS/MAPK/ERK and PI3K/Akt signaling pathways in LO2 cells after treated with recombinant *Cs*GRN protein and inhibitors. Quantification of protein was measured by Image J and showed under each band. ^*^ *P* < 0.05 *versus* control group, ^**^ *P* < 0.01 *versus* control group, ^***^ *P* < 0.001 *versus* control group, ^****^ *P* < 0.0001 *versus* control group.

**Figure 8:**
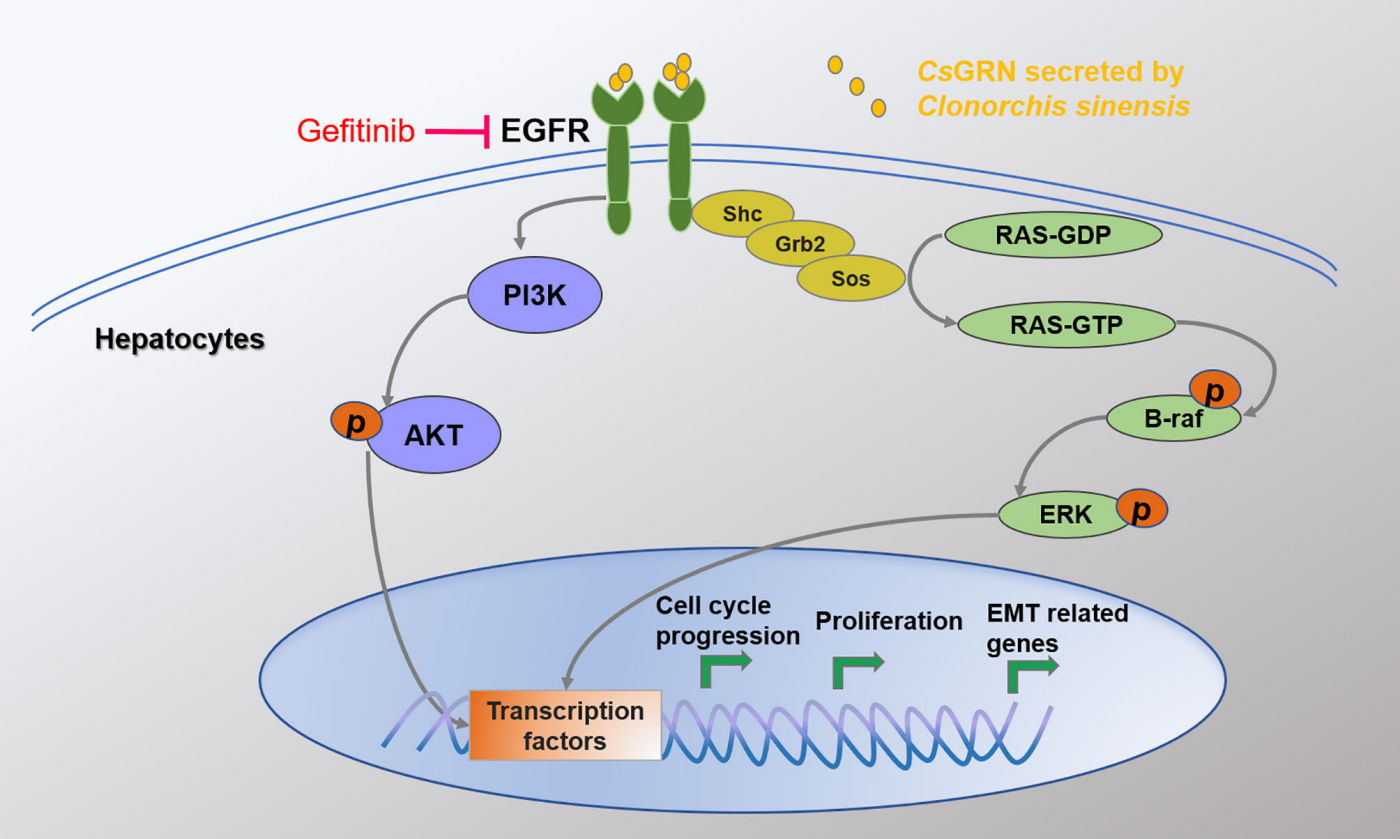
The mechanism of CsGRN in promoting malignant transformation of hepatocyte.

To identify the key role of EGFR in *Cs*GRN-induced hepatocytes’ malignant transformation, we co-cultured recombinant *Cs*GRN with EGFR inhibitors in LO2 cells. We found EGFR inhibitor could recover recombinant *Cs*GRN-induced cell proliferation, migration, and invasion of LO2 cells (**Figures 7A–C**). In addition, treating LO2 cell with EGFR inhibitor, Akt inhibitor, RAS inhibitor, braf inhibitor, and ERK inhibitor, respectively, could block the activation of RAS/MAPK/ERK and PI3K/Akt pathways, which were enhanced by recombinant *Cs*GRN (**Figure 7D**, **Figure S2**). Therefore, we could draw the conclusion that secreted *Cs*GRN protein promoted malignant transformation of hepatocytes by activation of RAS/MAPK/ERK and PI3K/Akt signaling pathways through EGFR (**Figure 8**).

## Discussion

*Clonorchis Sinensis* has a long history of recurrent infection and high intensity of infection, and it is an important risk factor for CCA and HCC (Shi et al., 2017). A total of 16.44% of HCC patients in China were infected with *Clonorchis Sinensis*, while the infection rate of non-tumor patients was 2.4%. Within the HCC group, the OR value and 95% CI were 8.00 and 4.34–14.92, respectively (Chen et al., 2013). It has been obvious that *Cs*ESPs secreted by liver flukes are involved in the pathogenesis of HCC (Chen et al., 2013). We previously showed that *Cs*GRN, as a homologue of a human growth factor, belongs to the granulin family, and it is an important component of *Cs*ESPs (Wang et al., 2011; Wang et al., 2014). What’s more, overexpression of *Cs*GRN protein enhanced the migration and invasion of HCC (Wang et al., 2017). It has been noted that granulin secreted by *O. viverrine* promotes the cellular proliferation of cholangiocytes and facilitates wound healing (Smout et al., 2015).

In the present study, we demonstrated that *Cs*GRN promoted the proliferation of LO2 cells by regulating cell-cycle-related genes. When being transfected with *Cs*GRN-overexpressing lentivirus plasmid, the expression of cell-cycle-related genes CyclinD1, CyclinD3, CDK2, CDK4, and CDK6 increased in LO2 cells, while the expression of p18, p21, and p27 decreased. Neoplastic transformation involves abnormal cell proliferation caused by dysregulating cell cycle progression (Fernandez et al., 2002). Most importantly, we found that the overexpressed *Cs*GRN also increased colony formation, invasion, and migration and induced EMT in LO2 cells. EMT plays a vital role in carcinogenesis, wound healing, and organ fibrosis (Arnoux et al., 2008; Thiery et al., 2009; Kriz et al., 2011; Carew et al., 2012). Our current findings revealed that the expression of vimentin, N-cadherin, and β-catenin protein as well as MMP9 mRNA significantly increased, while the expression of E-cadherin and ZO-1 protein dramatically decreased in the overexpressed *Cs*GRN groups, compared with the control groups. Those results indicated that *Cs*GRN regulated the migration and invasion of LO2 cells by inducing EMT in normal hepatocytes. The role of epithelial plasticity in HCC has become more and more prominent, since inducers of EMT such as transforming growth factor (TGF-β) can drive both fibrogenesis and carcinogenesis with rising cytokine levels in cirrhosis as well as late-stage HCC (Giannelli et al., 2016). Therefore, we confirmed that *Cs*GRN contributed dramatically to the malignant transformation of normal hepatocytes, which indicated that *Cs*GRN could serve as the crucial cancerogenic factor of HCC induced by *Clonorchis sinensis* infection. However, it still remains to be determined how *Cs*GRN affects malignant transformation in LO2 cells and its mechanism in regulating EMT.

To further elucidate the mechanism of *Cs*GRN-induced malignant transformation of normal hepatocytes, we investigated the epidermal growth factor receptor (EGFR)-mediated RAS/MAPK/ERK and PI3K/Akt signaling pathways. EGFR belongs to receptor tyrosine kinases (RTKs) of the ErbB family, and it is involved in the control of cell survival, growth, proliferation, and differentiation by extracellular signals (such as growth factors and cytokines) (Sigismund et al., 2018). When the ligand connects to the extracellular binding domain of EGFR, the phosphorylated tyrosine site of EGFR acts as the docking site for various proteins that are involved in the activation and regulation of signal transduction cascades (Morandell et al., 2008), including RAS/MAPK and PI3K/Akt pathways. It has been reported that overexpressed granulin protein in liver cancer can activate the ERK and PI3K/AKT signaling pathways and promote the growth, invasion, and metastasis of HCC (Cheung et al., 2004). Meanwhile, activated MAPK/ERK pathway would upregulate the expression of EGFR, thus forming an autocrine loop that promotes the growth of tumor cells and plays a positive feedback role (Anand et al., 2011). In our present study, we observed that both the mRNA and protein expression of p-EGFR, RAS, p-ERK, p-AKT, p-PI3K, and p-braf were dynamically upregulated in the overexpressed *Cs*GRN groups, which suggested that the overexpression of *Cs*GRN induced malignant transformation of normal hepatocytes *via* EGFR-regulated RAS/MAPK/ERK and PI3K/Akt signaling pathways. However, we did not investigate those findings *in vivo*, and future study will conduct the nude mouse xenograft assays to explore the role of *Cs*GRN during malignant transformation.

In conclusion, we provided evidences demonstrating that overexpression of *Cs*GRN induced the malignant transformation of LO2 hepatocytes by promoting cell proliferation, migration, and invasion *via* cell cycle arrest and EMT. Mechanistically, activated EGFR-mediated RAS/MAPK/ERK and PI3K/Akt signaling pathways were involved in these activities. Those findings proved that *Cs*GRN could play a crucial role in the malignant transformation of hepatocytes induced by *Clonorchis sinensis* infection. Our data provided valuable insights into the complex role of *Cs*GRN in *Clonorchis sinensis* infection–induced tumor biological activity.

## Data Availability Statement

The original contributions presented in the study are included in the article/**Supplementary Material**. Further inquiries can be directed to the corresponding authors.

## Ethics Statement

All experimental procedures regarding human cell lines were approved by the Research Ethics Board of Zhongshan School of Medicine in Sun Yat-sen University.

## Authors Contributions

CW, YW, and XL conceived and designed the experiments. CW and QH performed the experiments. CW, QH, YW, and YY analyzed the data. CW, XL, and QH wrote the manuscript. All authors contributed to the article and approved the submitted version.

## Funding

This work was supported by the Natural Science Foundation of Guangdong Province (Grant no. 2019A1515010583), the National Key R&D Program of China (Grant no. 2020YFC1200100), and the National Natural Science Foundation of China (Grant no. 81641094) to XL, the Postdoctoral Start-up Fund supported by the Sixth Affiliated Hospital of Sun Yat-Sen University (Grant No. R2021021720212991) to CW, and the Fundamental Research Funds for the Central Universities (Grant no. 20ykpy158) to YW.

## Conflict of Interest

The authors declare that the research was conducted in the absence of any commercial or financial relationships that could be construed as a potential conflict of interest.

## Publisher’s Note

All claims expressed in this article are solely those of the authors and do not necessarily represent those of their affiliated organizations, or those of the publisher, the editors and the reviewers. Any product that may be evaluated in this article, or claim that may be made by its manufacturer, is not guaranteed or endorsed by the publisher.

## Abbreviations

*Cs*GRN, *Clonorchis sinensis* granulin; ESPs, excretory/secretory products; HCC, hepatocellular carcinoma; CCA, cholangiocarcinoma; PDAC, pancreatic ductal adenocarcinoma; PGRN, progranulin; MMPs, matrix metalloproteinases; MMP9, matrix metalloproteinase 9; EMT, epithelial to mesenchymal transition; EGFR, epidermal growth factor receptor; ERK, extracellular signal-regulated kinase; AMPK, adenosine monophosphate-activated protein kinase; PI3K, phosphatidyl inositol 3-kinase.
