# Supplementary material for: Clonorchis sinensis Granulin Promotes Malignant Transformation of Hepatocyte Through EGFR-Mediated RAS/MAPK/ERK and PI3K/Akt Signaling Pathways
Source: Front Cell Infect Microbiol. 2021 Nov 10;11:734750. doi: 10.3389/fcimb.2021.734750 (PMC8631275; doi:10.3389/fcimb.2021.734750)
Supplement: Supplementary file 1 [file DataSheet_1.docx]

**Supplementary Data**

| **Figure S1.** The protein expression quantification of cell cycle markers measured by Image J |
| --- |
| **Figure S2.** Quantification of non-phosphorylated and phosphorylated forms of protein expression in signaling pathway measured by Image J |
| **Supplementary Table.** Primer pair sequences of human genes used in RT-qPCR |

**Figure S1**


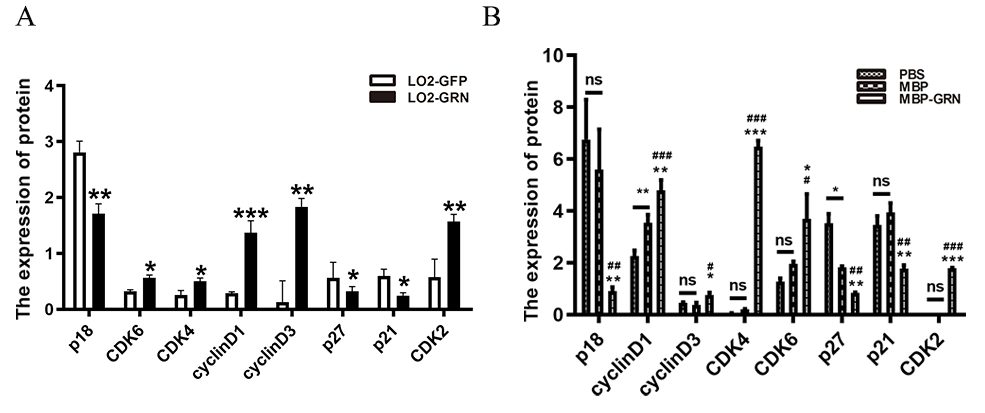


**Figure S1. The protein expression quantification of cell cycle markers measured by Image J.** (A) Overexpression of *Cs*GRN in LO2 cells. (B) LO2 cells co-cultured with recombinant *Cs*GRN with MBP tag.

**Figure S2**


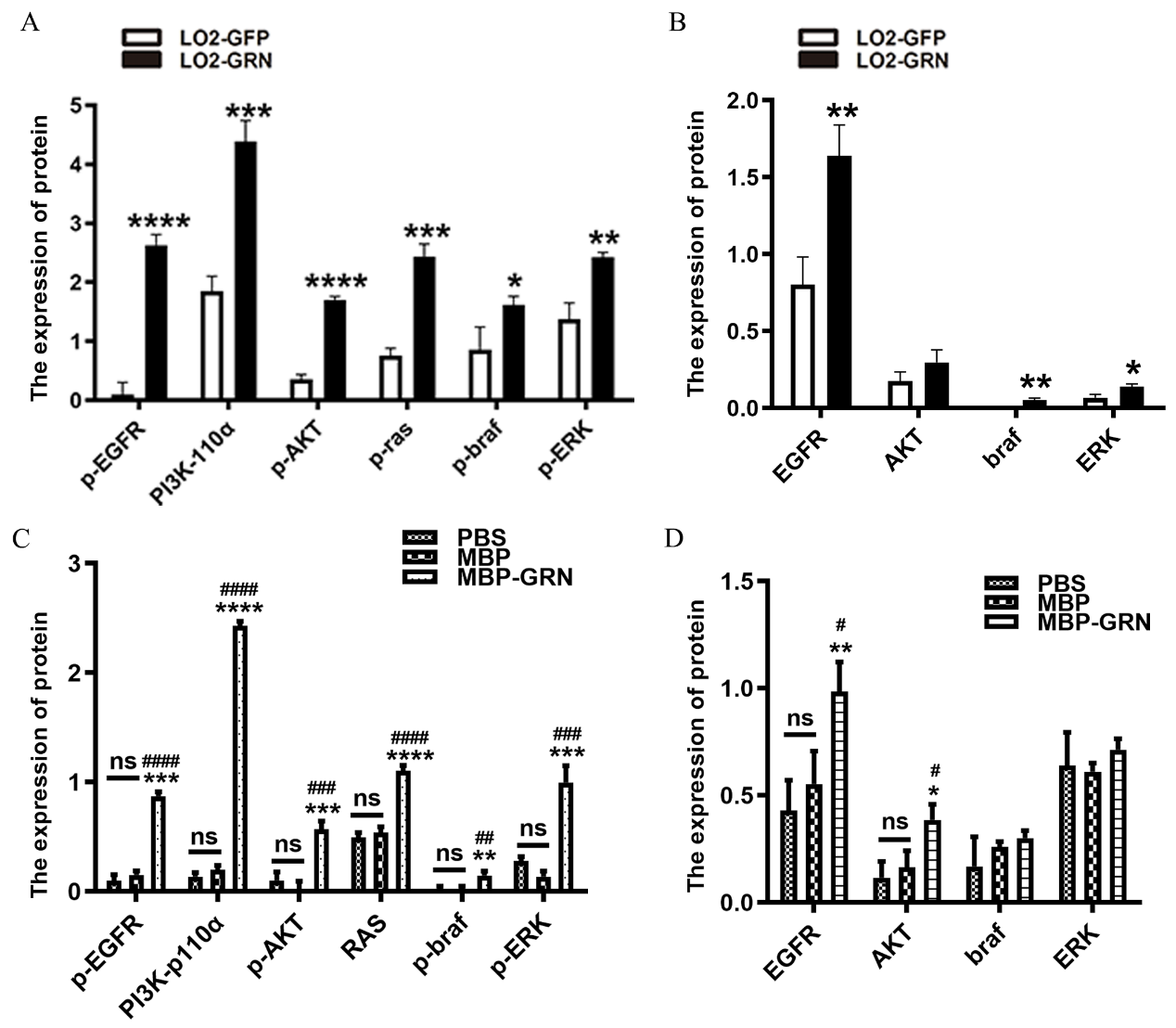


**Figure S2. Quantification of non-phosphorylated and phosphorylated forms of protein expression in signaling pathway measured by Image J.** (A, B) Overexpression of *Cs*GRN in LO2 cells. (C, D) LO2 cells co-cultured with recombinant *Cs*GRN with MBP tag.

**Supplementary Table. Primer pair sequences of human genes used in RT-qPCR**

| Genes | Forward primer sequence | Reserve primer sequence |
| --- | --- | --- |
| *Cs*GRN | 5'-CGCGGATCCTGTAAATATAACCAGACTTG-3' | 5'-TTACTCGAGCGGAGCACAGGTGTAGTGAT-3' |
| MMP9 | 5'-TGTACCGCTATGGTTACACTCG-3' | 5'-GGCAGGGACAGTTGCTTCT-3' |
| β-actin | 5'-GGCACTCTTAGCCTTCCTTCCT-3' | 5'-GCCAGACAGCACTGTGTTGGCGT-3' |
| p18 | 5'-CCACCTCCCTGTGGTGGAGT-3' | 5'-GTGGCTCCCCCAACCCCATT-3' |
| p21 | 5'-TGTCCGTCAGAACCCATGC-3' | 5'-AAAGTCGAAGTTCCATCGCTC-3' |
| p27 | 5'-AGGAGGAGATAGAAGCGCAGA-3' | 5'-GTGCGGACTTGGTACAGGT-3' |
| CDK2 | 5'-CCAGGAGTTACTTCTATGCCTGA-3' | 5'-TTCATCCAGGGGAGGTACAAC-3' |
| CDK4 | 5'-ATGGCTACCTCTCGATATGAGC-3' | 5'-CATTGGGGACTCTCACACTCT-3' |
| CDK6 | 5'-GCTGACCAGCAGTACGAATG-3' | 5'-GCACACATCAAACAACCTGACC-3' |
| CyclinD1 | 5'-GCTGCGAAGTGGAAACCATC-3' | 5'-CCTCCTTCTGCACACATTTGAA-3' |
| CyclinD3 | 5'-TACCCGCCATCCATGATCG-3' | 5'-AGGCAGTCCACTTCAGTGC-3' |
| EGFR | 5'-AGGCACGAGTAACAAGCTCAC-3' | 5'-ATGAGGACATAACCAGCCACC-3' |
| PI3K | 5'-CTGCCTGCGACAGATGAGTG-3' | 5'-TCCGATTACCAAGTGCTCTTTC-3' |
| AKT | 5'-TCCTCCTCAAGAATGATGGCA-3' | 5'-GTGCGTTCGATGACAGTGGT-3' |
| RAS | 5'-ACAGAGAGTGGAGGATGCTTT-3' | 5'-TTTCACACAGCCAGGAGTCTT-3' |
| p-braf | 5'-TGGGGAACGGAACTGATTTTTC-3' | 5'-TTTTGTGGTGACTTGGGGTTG-3' |
